# Novel lipid parameters for predicting and interpreting the severity of coronary artery lesions in premature coronary artery disease

**DOI:** 10.3389/fcvm.2026.1745711

**Published:** 2026-03-10

**Authors:** Hui Song, Qiang Geng, Yaowen Xu, Ying Ma, Jie Gao, Xiaowei Sun, Kang Zhang, Yongjie Yan, Fangjie Hou

**Affiliations:** 1 School of Clinical Medicine, Shandong Second Medical University, Weifang, China; 2 Department of Cardiology, University of Health and Rehabilitation Sciences (Qingdao Municipal Hospital), Qingdao, China

**Keywords:** Gensini score, nomogram, novel lipid parameters, prediction model, premature coronary artery disease

## Abstract

**Objective:**

To evaluate the predictive value of novel lipid parameters for coronary lesion severity in pCAD and to develop a nomogram-based prediction model.

**Methods:**

Patients newly diagnosed with pCAD at Qingdao Municipal Hospital (2021–2024) were enrolled and randomly assigned to training and validation cohorts in a 7:3 ratio. Coronary lesion severity was assessed using the Gensini score (GS), with patients stratified into mild or significant stenosis groups. Spearman correlation analysis was performed between GS and lipid parameters. Key predictors were selected using LASSO regression, and independent risk factors were identified by multivariable logistic regression to construct the nomogram model. The model's discrimination, calibration, and clinical utility were evaluated using receiver operating characteristic (ROC) curves, calibration plots, and decision curve analysis (DCA).

**Results:**

Lp(a), non-HDL-C, RC, FFA, and BAR were positively correlated with GS (r = 0.34, 0.34, 0.18, 0.19, 0.18; all *P* < 0.01). Lp(a) (OR = 1.03, *P* < 0.05), male sex (OR = 2.22, *P* < 0.05), FFA (OR = 2.40, *P* < 0.05), and non-HDL-C (OR = 2.07, *P* < 0.05) were independent risk factors for significant coronary artery stenosis. The nomogram model developed based on these variables demonstrated excellent predictive performance, with AUC values of 0.815 and 0.839 in the training and validation cohorts, respectively (*P* < 0.001).

**Conclusion:**

The proposed nomogram provides an effective tool for identifying pCAD patients with severe coronary artery stenosis, demonstrating robust predictive accuracy and potential clinical utility.

## 1 Introduction

Patients with Premature Coronary Artery Disease (pCAD) have a poor long-term prognosis, high recurrence risk, and frequently experience adverse outcomes, imposing a heavy burden on families and society. A literature review indicates that current pCAD risk factor studies predominantly focus on traditional cardiovascular risk factors, with limited large-scale screening studies. Consequently, there is an urgent clinical need for rapid, accurate, and simple methods to identify high-risk pCAD populations early, thereby enabling health guidance and effective intervention to reduce their incidence.

Studies have indicated that dyslipidemia, particularly elevated low-density lipoprotein cholesterol (LDL), is an independent risk factor for pCAD (1). However, existing evidence suggests that even with intensive lipid-lowering therapy to achieve LDL targets, patients still face elevated risks of major adverse cardiovascular events(MACE) (2). This risk is defined as “residual cardiovascular risk.” The ESC 2024 (3) Consensus highlights that, beyond controlling traditional markers, including LDL, attention should also be directed toward emerging parameters, including lipoprotein(a) [Lp(a)], non-high-density lipoprotein cholesterol (non-HDL-C), remnant cholesterol (RC), free fatty acids (FFA), and high-sensitivity C-reactive protein.

Previous studies have demonstrated associations between Lp(a), RC, non-HDL-C, and FFA and pCAD (4–8). However, the relationship between these novel lipid parameters and coronary lesion severity in pCAD remains unclear. This study aimed to evaluate the predictive value of novel lipid parameters to assess coronary lesion severity in patients with pCAD.

## 2 Materials and methods

### 2.1 Study participants and clinical parameters

This retrospective study enrolled 566 patients with newly diagnosed pCAD at the Department of Cardiology, Qingdao Municipal Hospital, between 2021 and 2024. The cohort was randomly divided into training and validation sets using a 7:3 ratio via computer-generated codes. Exclusion criteria included: (1) prior coronary angiography with percutaneous coronary intervention or coronary artery bypass grafting; (2) no previous use of antiplatelet and lipid-lowering drugs or irregular use and no use in the past 1 months; (3) Concurrent severe cardiac disease, malignancy, immune system disorders, hematologic disorders, severe hepatic or renal insufficiency, persistent inflammatory state, familial hypercholesterolemia, arteritis, or incomplete clinical data. This study was approved by the Ethics Committee of Qingdao Municipal Hospital (Ethics Approval No.: 2025-KTLL-020) with informed consent waived.

Collect general patient information upon admission (name, gender, age, smoking history, history of hypertension, history of diabetes, family history, height, and weight). Fasting blood samples were collected within 24 h of admission. All laboratory measurements—including Lp(a) (reported in mg/dL and measured by immunoturbidimetry), total cholesterol (TC), HDL-C, LDL-C, apolipoprotein B (ApoB), apolipoprotein A1 (ApoA1), non-HDL-C, RC, small dense LDL cholesterol (sd-LDL-C), the apolipoprotein B/A1 ratio (BAR), and FFA—were obtained before the administration of any anti-inflammatory, antiplatelet, or lipid-lowering medications. All lipid-related concentrations are expressed in mmol/L unless otherwise noted.

### 2.2 Definitions

pCAD (9): (1) men <55 years, women <65 years. (2) Obstructive stenosis ≥ 50% in the lumen diameter of any major coronary artery (including the left main coronary artery, left anterior descending artery, left circumflex artery, and right coronary artery) or their main branches.

Gensini score (10): The total score was calculated based on the Gensini scoring guidelines, which assess the pCAD severity. The Gensini score for each patient was independently assessed by at least two interventional cardiologists, who were blinded to other patient clinical data, to obtain the average score (Table 1). According to the median Gensini score, a score of <32 was defined as mild pCAD (Group I), and a score of ≥32 was defined as severe pCAD (Group II).

**Table 1:** Correspondence table of coronary artery lesion severity and weight coefficients.

Stenosis severity	Score (points)	Lesion location	Weight coefficient
1%–25%	1	Left main coronary artery	5
26%–50%	2	Proximal segment of the left anterior descending artery, proximal segment of the circumflex artery	2.5
51%–75%	4	Mid-segment of the left anterior descending artery	1.5
76%–90%	8	Distal segment of left anterior descending artery, mid segment of circumflex artery, distal segment of circumflex artery	1
91%–98%	16	Posterolateral branch of the left ventricle, obtuse marginal artery, first diagonal branch	1
99%–100%	32	Right coronary artery	1
		Other small branches	1

### 2.3 Statistical analysis

Statistical analysis was performed using SPSS 25.0 and R software version 4.51. Normally distributed quantitative data are presented as x ± s, with intergroup comparisons conducted using *t*-tests. Non-normally distributed quantitative data are presented as [M(Q1, Q3)], with intergroup comparisons conducted using the Mann–Whitney *U*-test. Categorical data are presented as frequencies (%), with intergroup comparisons conducted using chi-square tests. LASSO regression with 10-fold cross-validation was used to select predictive variables. Multicollinearity among the independent variables was assessed using the variance inflation factor(VIF). A value lower than 5 was considered to indicate no severe multicollinearity. The selected variables were used to construct a multivariate logistic regression model on the training set. To assess the influence of potential unmeasured confounding (incompletely documented medication history) on the study findings, we calculated E-values for the primary predictors. Based on the regression results, a nomogram prediction model was developed. The model was evaluated using ROC curves, calibration curves, and DCA. Statistical significance was set at *P* < 0.05.

## 3 Results

### 3.1 Clinical data of patients

The baseline clinical characteristics and laboratory test results of patients with pCAD in the different GS score groups are presented in Table 2. A total of 566 patients were enrolled in this study and divided into two groups based on GS scores: GS I (256 patients) and GS II (310 patients). No statistically significant differences were observed between the two groups regarding family history of premature coronary heart disease, hypertension, diabetes history, body mass index (BMI), TC, LDL, ApoB, or sd-LDL-C levels (*P* > 0.05). Statistically significant differences were observed between the two groups in terms of gender, age, smoking history, Lp(a), HDL, non-HDL-C, RC, ApoA1, BAR, and FFA levels (*P* < 0.05).

**Table 2:** Clinical baseline data statistics.

Characteristic	All pCAD participants (*n* = 566)	Group Ⅰ (*n* = 256)	Group Ⅱ (*n* = 310)	*P* value
Male, *n* (%)	346 (61.1)	130 (50.8)	216 (69.7)	***<0***.***001***
Age, years	52.2 ± 7.5	53.4 ± 6.9	51.2 ± 7.8	***<0***.***001***
Smoking, *n* (%)	241 (42.58)	87 (34.0)	154 (49.7)	***<0***.***001***
Hypertension, *n* (%)	367 (64.84)	165 (64.5)	202 (65.2)	0.861
Family history of CAD, *n* (%)	68 (12.01)	34 (6.01)	34 (6.01)	0.260
Diabetes, *n* (%)	165 (29.15)	68 (26.6)	97 (31.3)	0.218
BMI (kg/m^2^)	26.86 ± 3.86	26.37 ± 3.71	26.66 ± 3.97	0.214
TC (mmol/L)	4.63 (3.85–5.47)	4.52 (3.83–5.36)	4.71 (3.87–5.49)	0.275
HDL (mmol/L)	1.03 (0.86–1.22)	1.05 (0.89–1.23)	1.02 (0.84–1.19)	***0***.***029***
LDL-C (mmol/L)	2.77 (2.21–3.36)	2.70 (2.14–3.26)	2.83 (2.24–3.42)	0.132
sd-LDL-C (mmol/L)	0.91 (0.68–1.10)	0.89 (0.68–1.10)	0.93 (0.69–1.11)	0.165
non-HDL-C (mmol/L)	3.65 (2.90–4.29)	2.93 (2.32–3.50)	3.96 (2.94–4.45)	***<0***.***001***
RC (mmol/L)	0.78 (0.58–1.01)	0.75 (0.54–0.91)	0.88 (0.64–1.12)	***<0***.***001***
ApoA1 (mmol/L)	1.21 ± 0.25	1.26 ± 0.26	1.14 ± 0.23	***<0***.***001***
ApoB (mmol/L)	1.03 (0.84–1.25)	1.00 (0.83–1.23)	1.06 (0.84–1.26)	0.179
BAR (mmol/L)	0.89 (0.68–1.10)	0.82 (0.64–1.02)	0.93 (0.72–1.15)	***<0***.***001***
FFA (mmol/L)	0.46 (0.33–0.61)	0.42 (0.30–0.56)	0.50 (0.34–0.64)	***0***.***003***
Lp(a) (mg/dL)	22.64 (10.33–43.51)	13.66 (8.20–26.45)	33.54 (15.37–52.83)	***<0***.***001***

Italic bold values are statistically significant. BMI, body mass index; TC, total cholesterol; HDL, high-density lipoprotein cholesterol; LDL, low-density lipoprotein cholesterol; sd-LDL-C, small dense low-density lipoprotein cholesterol; non-HDL-C, non-high-density lipoprotein cholesterol; RC, remnant cholesterol; ApoA1, apolipoprotein A1; ApoB, apolipoprotein B; BAR, apolipoprotein B/apolipoprotein A1; FFA, free fatty acid; Lp(a), lipoprotein(a); GS, Gensiniscore.

### 3.2 Correlation analysis between GS scores and blood lipid parameters

**Figure 1:**
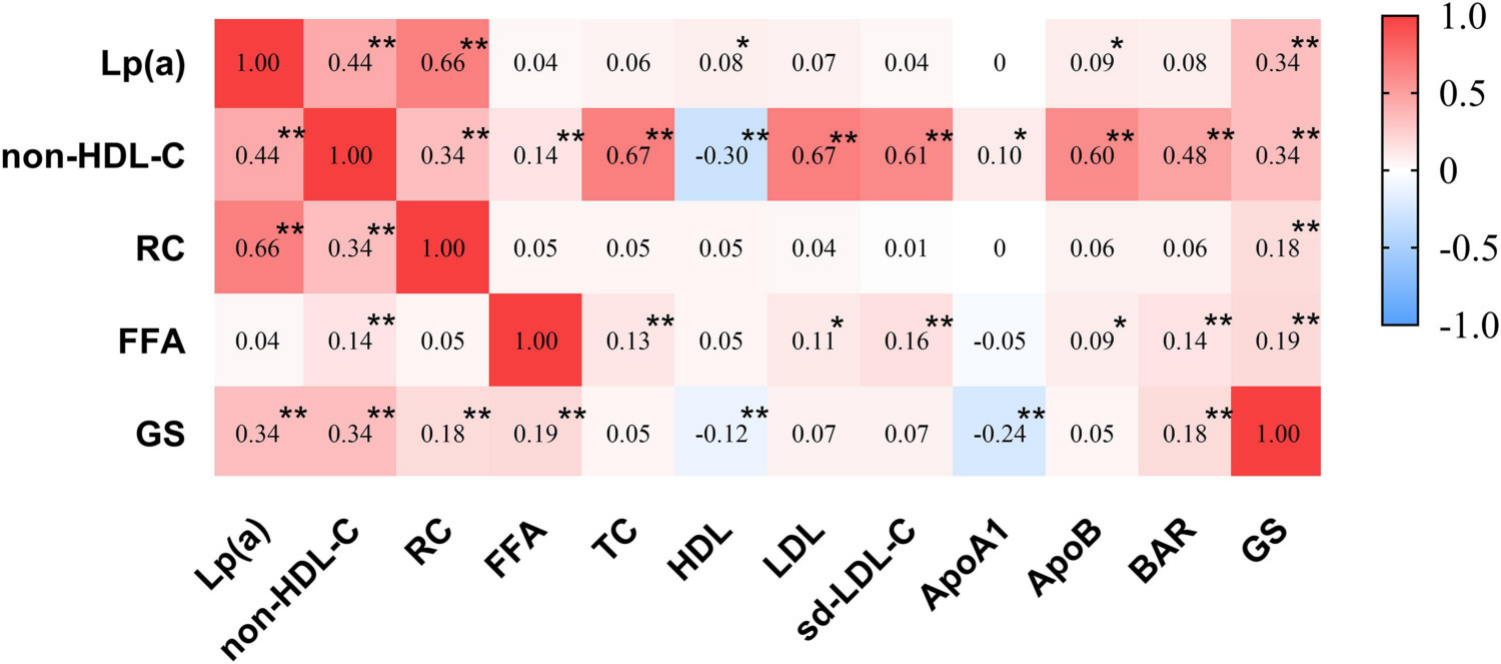
Heatmap of correlations between lipid parameters and GS scores. Spearman correlations between Gensini score and traditional lipid parameters. Cells are highlighted with ‘*’ when meeting the *P*-value threshold of *P* *<* *0.05*. Cells are highlighted with ‘**’ when meeting the *P*-value threshold of *P* *<* *0.01*. TC, total cholesterol; HDL, high-density lipoprotein cholesterol; LDL, low-density lipoprotein cholesterol; sd-LDL-C, small dense low-density lipoprotein cholesterol; non-HDL-C, non-high-density lipoprotein cholesterol; RC, remnant cholesterol; ApoA1, apolipoprotein A1; ApoB, apolipoprotein B; BAR, apolipoprotein B/apolipoprotein A1; FFA, free fatty acid; Lp(a), lipoprotein(a); GS, Gensiniscore.

Spearman correlation analysis revealed that the GS score was associated with the lipid parameters Lp(a), HDL, non-HDL-C, RC, ApoA1, FFA, and BAR, with correlation coefficients of r = 0.34, −0.12, 0.34, 0.18, −0.24, 0.19, and 0.18, respectively (all *P* < 0.05) (Figure 1).

### 3.3 Construction of the risk factor model

**Figure 2:**
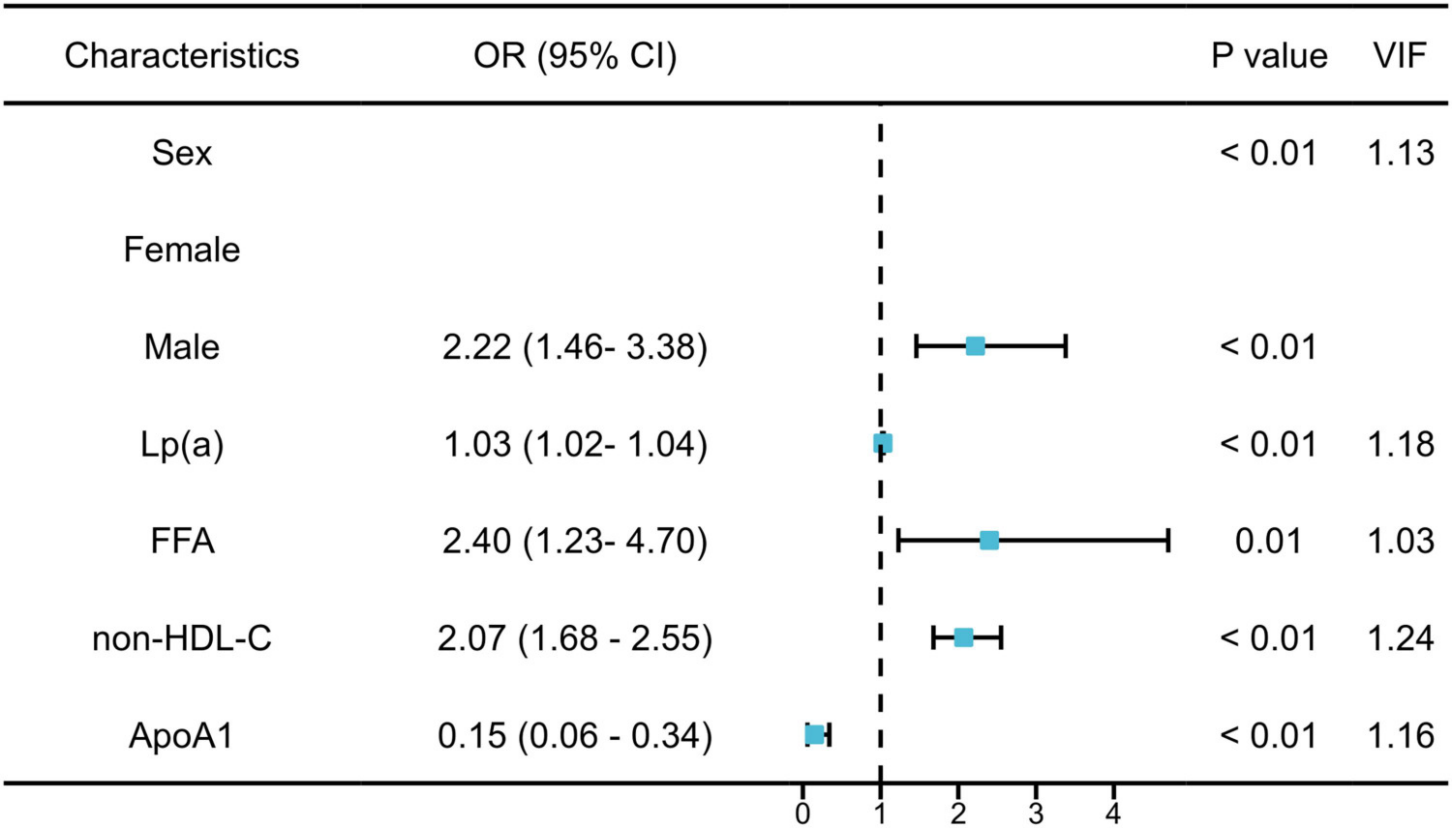
Multivariate logistic regression analysis of the severity of coronary artery stenosis in pCAD and VIF. FFA, free fatty acid; Lp(a), lipoprotein(a); non-HDL-C, non-high-density lipoprotein cholesterol.

LASSO regression was first applied, followed by backward stepwise selection, which identified five optimal predictors from an initial set of 18 variables significantly associated with significant coronary artery stenosis in pCAD patients: gender, Lp(a), FFA, non-HDL-C, and ApoA1. Subsequent multivariable logistic regression analysis confirmed Lp(a), FFA, non-HDL-C, and male gender as independent risk factors for significant stenosis, while ApoA1 was identified as an independent protective factor (all *P* < 0.05) (Figure 2). The E-value for the significant association between FFA and coronary stenosis was 2.46, with an E-value for the lower limit of the 95% CI of 1.45. This indicates that to explain away the observed association, an unmeasured confounder would need to be associated with both FFA and coronary stenosis by risk ratios of at least 2.46 (most conservatively, 1.45). Similarly, the E-values for Lp(a) and non-HDL-C were 1.13 and 2.23, respectively.

### 3.4 Construction of the nomogram prediction model

**Figure 3:**
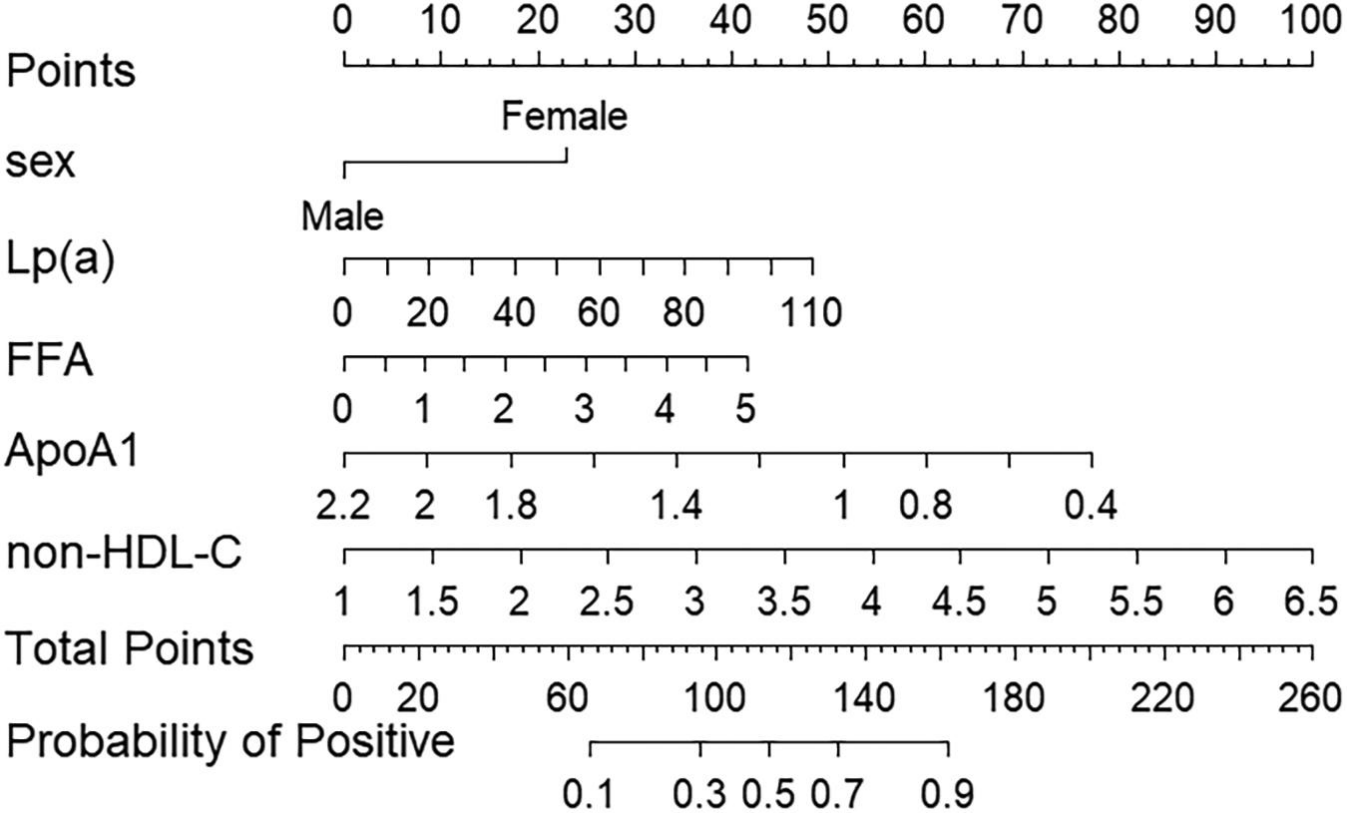
Nomogram for predicting the risk of severe coronary artery lesions in pCAD. FFA, free fatty acid; Lp(a), lipoprotein(a); non-HDL-C, non-high-density lipoprotein cholesterol.

Based on the multivariable logistic regression results, a nomogram was developed to quantitatively predict the probability of significant coronary artery stenosis in patients with premature coronary artery disease (pCAD). Multidisciplinary among predictors was assessed using the variance inflation factor (VIF); all variables showed VIF < 5 (Figure 2). The nomogram incorporated gender, Lp(a), FFA, non-HDL-C, and ApoA1 (Figure 3). Each variable contributes a point score; the sum of these scores corresponds to an individual's estimated risk of significant stenosis.

### 3.5 Verification of the nomogram prediction model

**Figure 4:**
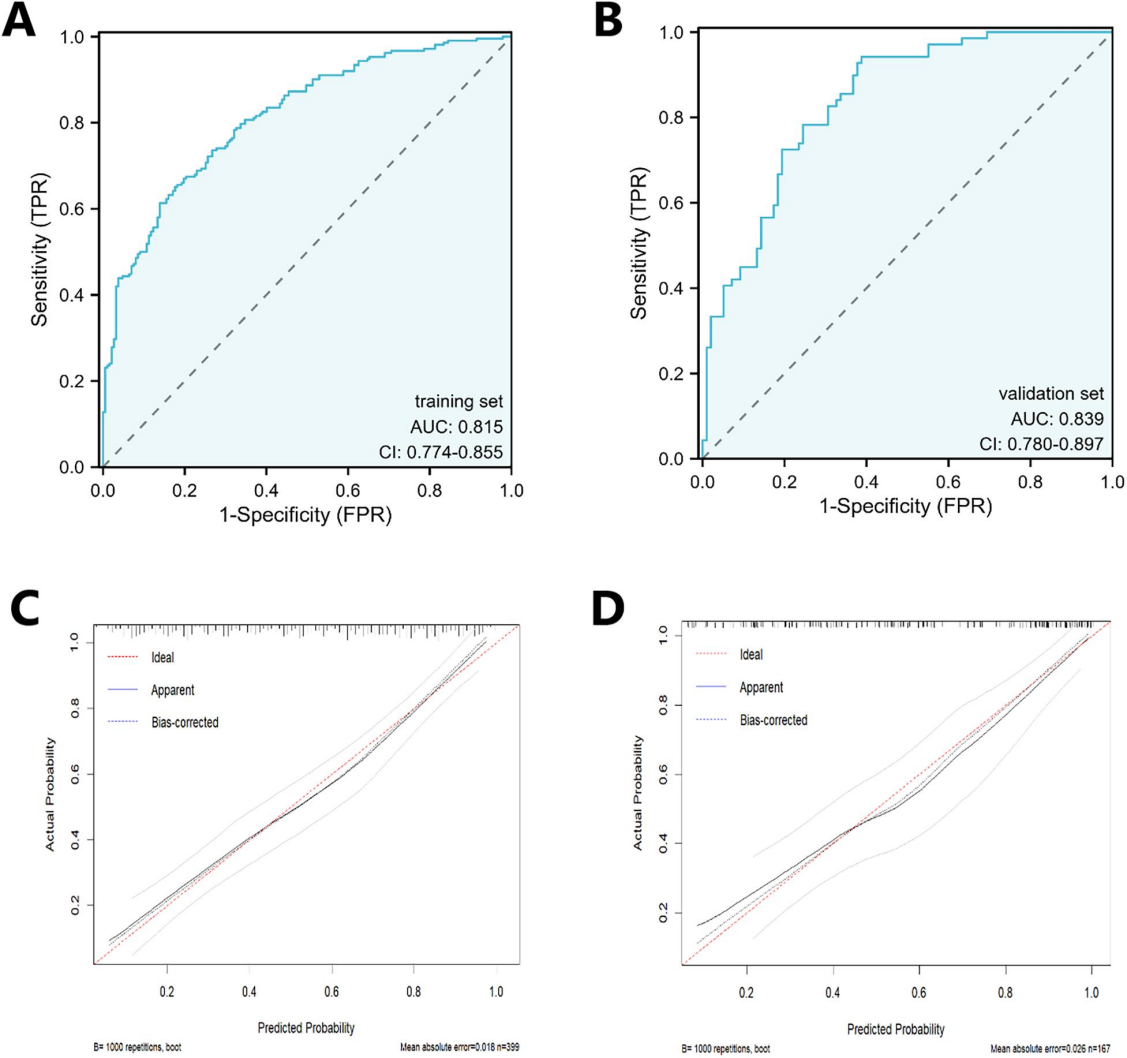
The ROC and calibration curve of the training set and the testing set. **(A)** is the ROC of the training set, and **(B)** is the ROC of the testing set. **(C)** is the calibration curve of the training set, and **(D)** is the calibration curve of the testing set.

**Figure 5:**
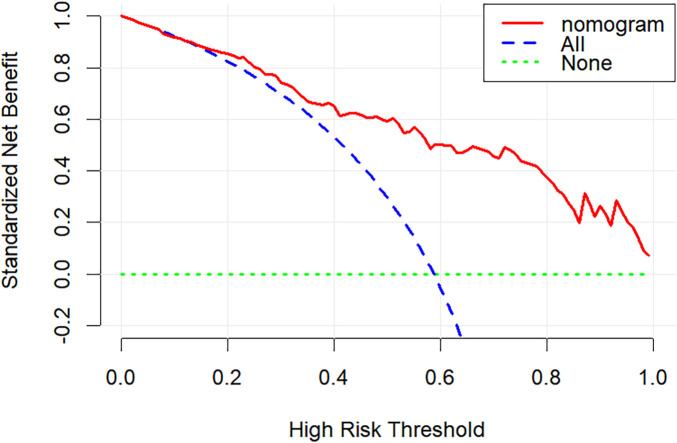
The DCA of the nomogram.

Model discrimination was evaluated using ROC curves. The area under the curve (AUC) was 0.815 in the training set and 0.839 in the validation set. Calibration curves demonstrated close agreement between predicted probabilities and actual outcomes in both sets, aligning well with the ideal diagonal (Figure 4). A good model fit was further supported by non-significant Hosmer-Lemeshow test results (*P* = 0.89 for training, *P* = 0.30 for validation). DCA demonstrated that the nomogram provided a substantial net clinical benefit across a wide range of threshold probabilities (Figure 5).

## 4 Discussion

This study demonstrates that Lp(a), FFA, male gender, and non-HDL-C are independent risk factors for significant coronary stenosis, with FFA presenting the highest risk. A nomogram incorporating these factors exhibited excellent predictive performance (AUC: 0.815 in training and 0.839 in validation), providing a tool for refined risk stratification and early intervention in pCAD management.

Our findings are substantiated by distinct pathophysiological mechanisms relevant to early-onset atherosclerosis. Lp(a) exerts a pronounced effect in pCAD through both genetic predisposition and multifaceted atherogenic actions (11, 12). Its plasma concentration is largely genetically determined, allowing its risk effect to manifest early in life, independent of accumulating environmental factors (12). Lp(a) carries proinflammatory oxidized phospholipids and exhibits high affinity for arterial wall components, directly promoting foam cell formation and plaque inflammation (13–15). Its structural homology with plasminogen inhibits fibrinolysis, tipping the balance toward a prothrombotic state (16). This combination of accelerated atherogenesis and impaired thromboresistance is particularly deleterious in younger patients.

FFA emerged as the strongest independent lipid risk factor in our cohort. Its pathophysiological role in pCAD may be linked to the early metabolic disturbances often observed in younger individuals. Elevated FFA levels directly induce endothelial dysfunction, a critical initiating event in atherosclerosis (17). Furthermore, FFA drives systemic insulin resistance and activates key inflammatory pathways, such as the NLRP3 inflammasome, within vascular cells (18). This creates a local and systemic proinflammatory environment that accelerates plaque initiation and progression, providing a mechanistic bridge between early metabolic dysfunction and premature coronary disease.

Non-HDL-C serves as an integrative measure of all atherogenic apolipoprotein B-containing particles, including LDL, triglyceride-rich lipoproteins (TRLs), their remnants, and Lp(a). This composite nature may explain its robust predictive value for cardiovascular events across diverse populations, including those with pCAD (19). Notably, the cholesterol content within TRLs and their remnant particles is reported to be highly atherogenic, with each particle carrying a substantial cholesterol load comparable to or exceeding that of LDL (20). This underscores that in aggressive, early-onset disease, the total burden of atherogenic cholesterol—captured by Non-HDL-C—is a critical determinant of risk, potentially more so than LDL alone.

The absence of a significant association between LDL and lesion severity in our pCAD cohort further highlights that the atherogenic drivers in early-onset disease may extend beyond the conventional LDL pathway. The pro-inflammatory, pro-thrombotic, and direct vascular toxicities of Lp(a), FFA, and RC create a synergistic vicious cycle that rapidly accelerates plaque development and instability in younger individuals.

Interestingly, while RC and the BAR ratio showed positive correlations with lesion severity in Spearman analysis, they were not independent risk factors in our multivariate model. This suggests that their association may be mediated by or confounded with established risk factors (e.g., age, gender, comorbidities) or other lipid parameters like Lp(a). RC can directly penetrate the vascular wall and bind to macrophage scavenger receptors without requiring oxidative modification (21), thereby accelerating foam cell formation and thrombus generation. Previous research indicates that certain lipid ratios may outperform traditional lipid markers (for example, LDL) in predicting cardiovascular event risk (17). A prospective cohort study (22) demonstrated that the BAR outperformed individual lipid markers in predicting coronary artery obstruction in patients with CAD undergoing percutaneous coronary intervention. Although not independently predictive in our cohort, these markers warrant further investigation into their mechanisms and conditional clinical utility.

## 5 Conclusion

In conclusion, this study identifies Lp(a), FFA, and non-HDL-C as key independent lipid risk factors for severe coronary stenosis in pCAD. The developed nomogram provides a clinically applicable tool for risk prediction. Although RC and BAR demonstrated correlated effects, their lack of independence necessitates cautious interpretation and further research. These findings support incorporating novel lipid parameters into the risk assessment paradigm for pCAD to enable more personalized management strategies.

### 5.1 Limitations

First, the severity of the coronary artery lesions was determined based on the operator's visual assessment of stenosis, introducing a degree of subjectivity. Although we attempted to control for the potential confounding effects of lipid-lowering and anti-inflammatory treatment history through strict exclusion criteria, as a retrospective study, residual confounding from unmeasured medication use cannot be fully excluded. Future prospective studies should systematically collect and document detailed medication histories during the design phase to more precisely evaluate the independent predictive value of these novel lipid parameters. Third, all participants in this study were recruited from Qingdao Municipal Hospital, representing a single-center retrospective design with limited sample representativeness. Future multicenter studies with larger sample sizes and rigorous control of confounding factors are required to validate these findings and enhance their reliability and applicability.

## Data Availability

The raw data supporting the conclusions of this article will be made available by the authors, without undue reservation.
